# A four‐dimensional dynamic conformal arc approach for real‐time tumor tracking: A retrospective treatment planning study

**DOI:** 10.1002/acm2.14224

**Published:** 2023-12-25

**Authors:** Timothy Yau, Jeff Kempe, Stewart Gaede

**Affiliations:** ^1^ Department of Medical Biophysics University of Western Ontario London Canada; ^2^ London Health Sciences Centre London Canada; ^3^ Lawson Health Research Institute London Canada

**Keywords:** DCA, dose calculation, dynamic tumor tracking, respiratory management, VMAT

## Abstract

**Purpose:**

For many thoracic tumors, patient respiration can introduce a significant amount of variability in tumor position that must be accounted for during radiotherapy. Of all existing techniques, real‐time dynamic tumor tracking (DTT) represents the most ideal motion management strategy but can be limited by the treatment delivery technique. Our objective was to analyze the dosimetric performance of a dynamic conformal arc (DCA) approach to tumor tracking on standard linear accelerators that may offer similar dosimetric benefit, but with less complexity compared to intensity‐modulated radiation therapy (IMRT) or volumetric modulated arc therapy (VMAT).

**Methods:**

Ten patients who previously received free‐breathing VMAT for lung cancer were retrospectively analyzed. Patient 4D‐CT and respiratory traces were simultaneously acquired prior to treatment and re‐planned with DCA and VMAT using the Eclipse v15.6 Treatment Planning System with gated, deep inspiration breath hold (DIBH), and motion encompassment techniques taken into consideration, generating seven new plans per patient. DTT with DCA was simulated using an in‐house MATLAB script to parse the radiation dose into each phase of the 4D‐CT based on the patient's respiratory trace. Dose distributions were normalized to the same prescription and analyzed using dose volume histograms (DVHs). DVH metrics were assessed using ANOVA with subsequent paired *t*‐tests.

**Results:**

The DCA‐based DTT plans outperformed or showed comparable performance in their DVH metrics compared to all other combinations of treatment techniques while using motion management in normal lung sparing (*p* < 0.05). Normal lung sparing was not significantly different when comparing DCA‐based DTT to gated and DIBH VMAT (*p* > 0.05), while both outperformed the corresponding DCA plans (*p* < 0.05). Simulated treatment times using DCA‐based DTT were significantly shorter than both gating and DIBH plans (*p* < 0.05).

**Conclusions:**

A DCA‐based DTT technique showed significant advantages over conventional motion encompassment treatments in lung cancer radiotherapy, with comparable performance to stricter techniques like gating and DIBH while conferring greater time‐saving benefits.

## 1 INTRODUCTION

Tumors located near the thorax and abdomen can experience a significant range of motion due to patient respiration. Thus, intrafraction motion represents a concern during free‐breathing radiotherapy due to the risk of the tumor moving in and out of high dose regions, potentially under‐dosing the tumor and overdosing surrounding healthy tissue. While surgery in the form of lobectomy remains the standard treatment for early‐stage, non‐small cell lung cancer (NSCLC), stereotactic body radiotherapy (SBRT) has shown promising outcomes in medically inoperable patients.^1^, ^2^ However, the hypofractionation scheme used increases the amount of dose delivered per fraction, increasing the risk for radiation‐induced toxicities such as pneumonitis and vascular injury,^3^, ^4^, ^5^ and further emphasizing the importance of motion management during lung radiotherapy.

The AAPM Task Group 76 report is currently the most comprehensive document outlining the various techniques, current and emerging, that are used to account for tumor motion during treatment delivery.^6^ Techniques such as respiratory gating^7^ and deep inspiration breath hold (DIBH)^8^ have been able to successfully decrease treatment margins in lung SBRT by attempting to minimize target motion during beam delivery. However, treatment times using these techniques can be several minutes longer compared to free‐breathing treatments due to the constant beam deactivation to compensate for patient respiration. Furthermore, both gated and DIBH techniques require patient compliance with respiratory coaching to ensure consistent performance during treatment delivery,^7^, ^9^ which can further increase clinical workflow complexity or disqualify certain patients from using these techniques.

Unlike gated and DIBH techniques, a motion encompassment approach to respiratory motion management attempts to account for tumor motion by enlarging treatment margins to the full range of tumor motion, allowing the patient to breathe freely during treatment delivery. An internal target volume (ITV) is defined as a combination of the visible tumor while considering the potential motion and deformation of the volume during treatment. Typically, an ITV is created either by taking the union of the gross tumor volume (GTV) at every respiratory phase of the patient's 4D‐CT or by directly contouring on the patient's free‐breathing CT.^10^ However, widening the field size inevitably increases the amount of dose delivered to the surrounding healthy tissue. Instead, a tumor tracking technique where the radiation beam follows the target in real time represents the ideal in motion management, allowing a near 100% duty cycle while minimizing treatment margins and allowing the patient to breathe freely during treatment.

Currently, there are four clinically implemented systems capable of real‐time tumor tracking: Accuray's Synchrony Respiratory Tracking system, implemented with their CyberKnife system and RadiXact tomotherapy system (Accuray Inc, Sunnyvale, USA), the VERO gimbaled‐head radiotherapy device (BrainLAB, Chicago, USA; Mitsubishi Heavy Industries Ltd, Tokyo, JP; and Kyoto University, Kyoto, JP), and the MRIdian hybrid magnetic resonance linear accelerator (ViewRay Inc, Ohio, USA). However, widespread adoption of these four systems remains limited in North America, with only around 150 centers being equipped with a CyberKnife or RadiXact unit and under 30 MRIdian units within North America compared to the nearly 4000 linear accelerator units currently used in Canada and the United States. Additionally, only 12 VERO systems were ever installed outside Japan before manufacturing was ended. In comparison, multi‐leaf collimators (MLCs) are standard beam‐shaping devices equipped on conventional linear accelerators, making dynamic multi‐leaf collimator (DMLC) tracking, where the MLC positions are adjusted to the patient breathing in real time, a more accessible modality for tumor tracking.^11^, ^12^

It then becomes important to identify what treatment technique can be best implemented within the constraints of a DMLC‐based tracking system. While VMAT^13^ has become a common technique for lung SBRT treatments due to its quick delivery time compared to other static field techniques, improved dose conformity, and superior outcomes,^14^, ^15^ the steep dose gradients and complex modulation of MLC shapes increase the risk of under‐dosing the GTV. A potential interplay effect between the tumor motion and MLC modulation has been reported to cause dose errors of up to 20%.^12^, ^16^, ^17^ Although, more recent studies have shown that VMAT treatments have an interplay effect within clinical tolerances, provided sufficient fractions or a wide enough target margin is used.^17^, ^18^, ^19^, ^20^ Due to the hypofractionation scheme used in SBRT treatments, there is a reduction in the averaging effect over each fraction, significantly decreasing target coverage and emphasizing the importance for sufficient target margins around the GTV in SBRT treatments.^17^, ^19^ However, a tracking technique aims to further reduce treatment margins, indicating a possible further risk for an interplay effect when considering a VMAT‐based tracking technique. This risk is only further compounded when considering the effect of complex MLC modulation with the inherent latency in tumor tracking prediction models.

Another drawback to using an inverse‐planning module, such as VMAT, is the increase in image data required for optimization due to moving from a 3D‐CT to a 4D‐CT dataset. A 4D optimization would require additional patient contours at each CT phase to use in the optimization as well as the ability to operate over a dynamic 4D dataset instead of a static 3D image, increasing the time spent at the planning step in the clinical workflow. To counteract this increase in workload, a simpler forward‐planned technique would minimize the amount of time spent on treatment planning. One such technique that has been considered as an alternative to VMAT for lung SBRT is dynamic conformal arc (DCA),^21^, ^22^, ^23^, ^24^ where treatment beams are rotated around the patient with a fixed dose rate and gantry speed while the MLCs conform directly to the target. Additionally, due to the uniform fluence to the target and open fields, the DCA plan would exhibit a decreased interplay effect, increasing its potential efficacy for implementation with a tracking technique. Studies have shown the performance of the DCA technique in non‐tracked lung SBRT has been varied, either showing inferior^23^ or comparable performance to VMAT^21^, ^22^, ^24^ when using an ITV approach for free‐breathing radiotherapy. Implementing DCA within a tracking framework could address these limitations and lead to comparable performance to VMAT while providing a simpler implementation for tracking.

The purpose of this study was to compare target coverage and critical organ sparing of simulated DMLC tracking plans using a DCA technique with other existing respiratory management techniques using DCA and VMAT. While DCA exhibits fewer degrees of freedom compared to VMAT, the reduced field margins when implemented with a tracking technique may still lead to significant dosimetric improvements over VMAT treated with conventional free breathing motion encompassment techniques.

## 2 METHODS

### 2.1 Data acquisition

After obtaining institutional ethics approval (Lawson Approval Number: R‐23‐086), ten (10) patients with early‐stage NSCLC treated with a hypofractionated treatment scheme using a free‐breathing VMAT technique were retrospectively analyzed in the treatment planning system Eclipse v15.6 (Varian Medical Systems, Palo Alto, USA). Seven patients were treated with an ablative dose defined by a BED_10_ > 100 Gy, while three were retreatment scenarios with a reduced dose per fraction. 4D‐CT and DIBH scans of all patients were acquired on a Philips Brilliance Big Bore CT Scanner (Philips Medical Systems, Cleveland, USA), with the 4D‐CT datasets binned into 10 phases labeled from 0% to 90% based on the portion of the respiratory cycle. The 0% phase corresponds to end‐inhale and the 50% phase corresponding to end‐exhale. Patient specific breathing traces were obtained simultaneously using the Varian Respiratory Gating for Scanners (RGSC) system (Varian Medical Systems, Palo Alto, USA). Patient and tumor characteristics are summarized in Table 1.

**TABLE 1:** Summary of patient demographic, treatment prescriptions, and tumor characteristics.

Patient no.	Sex	Tumor location	ITV Volume (cc)	End‐exhale GTV volume (cc)	Respiratory amplitude (mm)	Treatment prescription (Gy/Fraction)
1	M	RLL	1.25	0.55	10.31	60 Gy/15
2	F	RLL	10.32	4.78	10.51	60 Gy/8
3	M	LUL	3.65	1.38	14.98	60 Gy/12
4	M	RML	8.46	6.20	13.57	60 Gy/15
5	M	RML	1.44	0.54	13.57	60 Gy/8
6	M	LLL	24.23	15.61	11.12	54 Gy/3
7	M	LUL	16.75	7.44	11.37	55 Gy/5
8	M	RLL	3.87	1.27	9.95	55 Gy/5
9	M	LUL	11.85	1.83	13.09	60 Gy/8
10	F	LUL	7.37	3.50	6.82	60 Gy/8

Abbreviation: GTV, gross tumor volume.

### 2.2 DCA tracking planning

GTVs were manually contoured on each respiratory phase of the 4D‐CT dataset for each patient. An isometric 5 mm expansion of the GTV on each phase was used to define a tracking planning target volume (PTV) on each phase. An initial DCA plan was created on the end‐exhale CT using the clinically delivered VMAT plan as a template, preserving the same prescription, energy (6MV or 6MV‐FFF), arc lengths, collimator angles, beam weights, and isocenters. Control points for the DCA plan were created using Eclipse v15.6 to conform the MLCs to the projection of the tracking PTV at each gantry angle with a 3 mm margin to account for the beam penumbra. A fixed dose rate of 1400 MU/min and a constant gantry speed were used throughout each arc for all plans. The DCA plan was normalized to the same 95% PTV coverage as its template VMAT plan by adjusting the gantry speed.

To simulate a tumor tracking dose calculation, we adapt a similar approach used by Rao et al.^25^ for computing 4D doses for arc‐based therapies. The patient's breathing trace was partitioned into a respiratory phase between 0% and 50% based on amplitude. Each DCA plan was exported from Eclipse as a DICOM file and an in‐house MATLAB (Mathorks, Massachusetts, USA) script was used to map each control point into the respiratory phase that the patient would be present in at that point during treatment delivery. However, control points located temporally near phase transitions can potentially contribute dose to multiple respiratory phases. To address this, the script would insert additional control points at each phase transition to partition the dose into each respiratory phase. In cases where the required delivery time is greater than the length of the available breathing trace, a portion of the prior trace with similar continuity was appended to extend the breathing trace by the required length.

**FIGURE 1:**
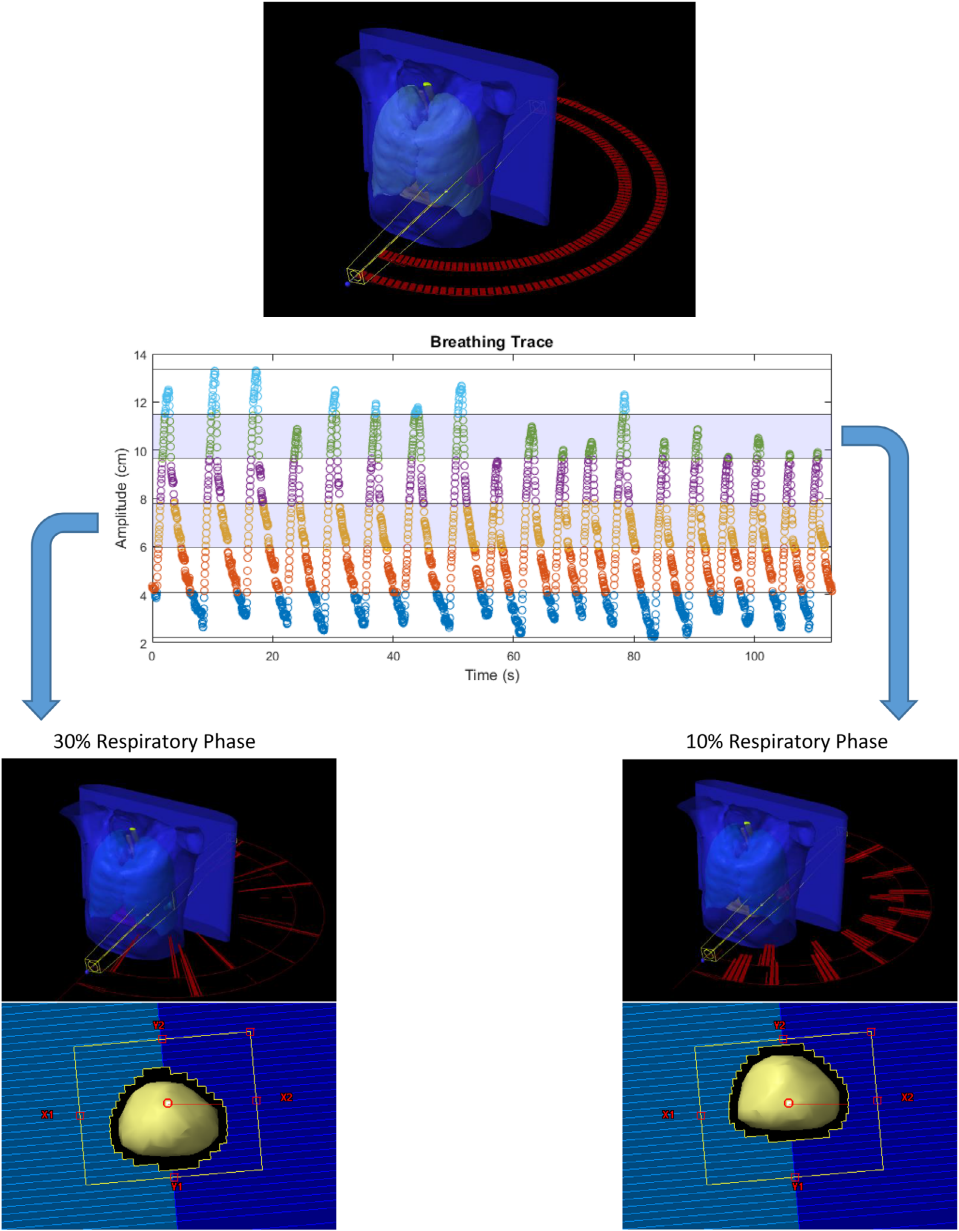
The tumor tracking dose calculation algorithm parses the beam arcs into each respiratory phase to calculate radiation delivery during a tracked plan at each phase. Beam delivery outside the specified phase is set to 0 while the MLC aperture conforms to the tumor's position at each phase to simulate tracking within the eclipse dose‐calculating environment. MLC, multi‐leaf collimators.

For each respiratory phase, the control points not corresponding to the specified phase had their monitor unit (MU) delivery set to 0 such that the output plan would simulate partial delivery of the DCA plan corresponding only to the selected phase. The modified plans for each respiratory phase were imported back into Eclipse and copied onto the corresponding CT phase. Eclipse could then conform the MLC to projections of the PTV with a 3 mm margin at each phase to simulate tumor tracking, as shown in Figure 1. Each phase‐parsed plan was recalculated in Eclipse using the Acuros XB algorithm and the dose matrices were imported into MIM (MIM Software Inc, Cleveland, USA) to accumulate the doses onto a single reference phase to account for non‐rigid deformations between the tumor and surrounding organs at risk (OARs). The end‐exhale phase is commonly selected as one of the more stable phases, and so was used as our reference phase. The resulting accumulated dose reflects a DCA‐based tumor tracking dose calculation.

### 2.3 Deformable image registration

For this study, the deformable image registration (DIR) and dose accumulation was performed using the Intensity‐Based Deformable Algorithm within the commercial software MIM.^26^ This DIR algorithm and dose accumulation has been verified extensively in the literature.^27^, ^28^, ^29^, ^30^ Guy et al. found that contours deformed across a thoracic 4D‐CT were clinically acceptable or only required minor revision 91.8% of the time.^27^ A multi‐institutional validation study of commercial DIR software conducted by Kadoya et al. found that the mean 3D registration error was 3.29 mm (2.17–3.61 mm).^28^ Mittauer et al. evaluated the dosimetric error in the dose accumulation using a heterogeneous, deformable, anthropomorphic phantom with implanted thermoluminescent dosimeters and found a median dose error of 0.6%, with a maximum difference of 0.31 Gy.^29^ Additionally, Nelli et al. analyzed the dosimetric error in a single phase 4D‐CT dose accumulation using gamma analysis and found a minimum passing rate of over 99% when considering up to 2 cm amplitude of movement.^30^

### 2.4 Motion encompassment planning

Each patient was treated using 1−2 coplanar arcs. Treatment planning was performed on the patient's free breathing CT, derived as an average of each phase from the patient's 4D‐CT. The superposition of the GTV at each phase was contoured on the free breathing CT to define the ITV and a further isometric 5 mm expansion was used to define the PTV. Patient VMAT plans were recalculated using the Acuros XB algorithm in Eclipse v15.6 and assessed for clinical acceptance prior to this analysis. A corresponding ITV‐based DCA plan was created with the MLCs conforming to the PTV with a 3 mm margin and normalized to the same 95% PTV coverage as the recalculated VMAT plan. The same workflow described in section B was then used to perform a 4D dose recalculation on both motion encompassment‐based treatment plans after normalization, but without modifying the MLC positions to simulate the effects of intrafraction breathing on the dose distribution.

### 2.5 Gated and DIBH planning

DCA and VMAT plans were created using the same arc lengths and collimator angles on the end‐exhale phase and DIBH scan to simulate delivery only during either full end‐exhalation for gating or deep inspiration for the DIBH plan. The PTV was defined as the GTV with an isometric 5 mm expansion. An additional 3–10 mm expansion was incorporated with the DCA plans to account for the beam penumbra and control for hotspots. Plans were normalized to the same 95% PTV coverage as the ITV‐optimized VMAT plan.

### 2.6 Treatment time calculation

Beam‐on times per fraction were calculated for each treatment plan using the gantry speed between each control point in the plan. For each control point, *i*, the max gantry speed of 4.8 deg/s is initially assumed, where the gantry would slow down if the total number of MUs delivered between two control points exceeded the max deliverable dose rate of 1400 MU/min. The new gantry speed would then be calculated with,

$$\;MU\;per\;degree_{i}=\frac{MU_{i}}{\Delta \theta_{i}}\;$$

$$\;gantry\;speed_{i}=\frac{1400\;MU/min}{MU\;per\;degree_{i}}\;$$

**FIGURE 2:**
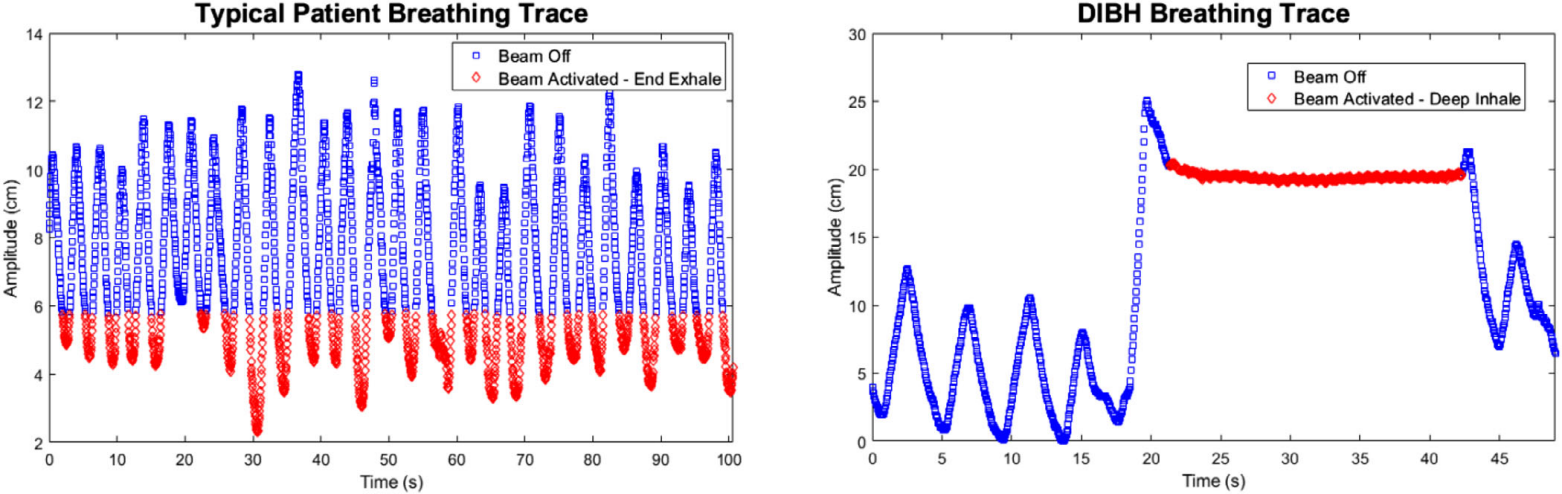
Sample respiratory traces for a patient in free breathing and breath hold. For gated and DIBH treatments, the radiation beam is only activated within a certain amplitude window shown in red on both traces. The total treatment time is calculated from the duration the patient spends within each beam‐on window. DIBH, deep inspiration breath hold.

For the tracking and motion encompassment plans, which are free‐breathing treatments, the beam‐on time is equivalent to the treatment time. For the gated and DIBH plans, the treatment time contains both beam‐on and beam‐off times to accommodate patient respiration. The gated plans were calculated by assuming the gating window would include the 40%−60% CT phases. DIBH delivery time was calculated using the patient breathing trace from their DIBH scan to derive the tolerable breath‐hold duration. The assumed required recovery time post breath‐hold was assumed to be equal to the breath‐hold duration. That is, DIBH treatment times were calculated assuming a 1:1 ratio of beam‐on time to recovery time for each breath‐hold. Example beam‐on windows for a simulated gated and DIBH delivery are illustrated in Figure 2.

### 2.7 Plan evaluation

Treatment plan performance was evaluated with various metrics from their dose volume histogram (DVH), with a better performance indicated by smaller dose‐volume metrics in the surrounding OARs. Individual DVH metrics were compared using repeated measures analysis of variance (ANOVA) and a Holm‐Bonferroni post‐hoc correction for multiple pairwise comparisons.^31^ The chosen DVH metrics for each ROI are summarized in Table 2. OAR sparing was evaluated with either D_95%_, D_2%_, or D_1%_, defined as the minimum dose received by at least 95%, 2%, or 1% of the structure respectively. The normal lung was defined as both lungs minus the GTV, with lung sparing evaluated using V_20Gy_, V_5Gy_, and mean dose, where V_20Gy_ and V_5Gy_ are the percent volume of the lung receiving at least 20 or 5 Gy respectively. PTV coverage was compared using D_95%_.

**TABLE 2:** DVH metrics used to compare plan performance in each ROI. Metrics are adapted from in‐house institutional dose constraints based on the ROSEL trial. Dose metrics are defined as the minimum dose received by the subscripted relative volume of the structure, while volume metrics are defined as the minimum relative volume receiving the subscripted dose.

ROI	DVH Metrics
PTV	D_95%_
GTV	D_95%_, D_1%_
Lungs	V_20Gy_, V_5Gy_, D_mean_
Heart	D_2%_
Spine	D_95%_, D_1%_
Esophagus	D_95%_, D_1%_

Abbreviations: DVH, dose volume histograms; GTV, gross tumor volume.

## 3 RESULTS

### 3.1 Dosimetric comparison of motion management techniques

**FIGURE 3:**
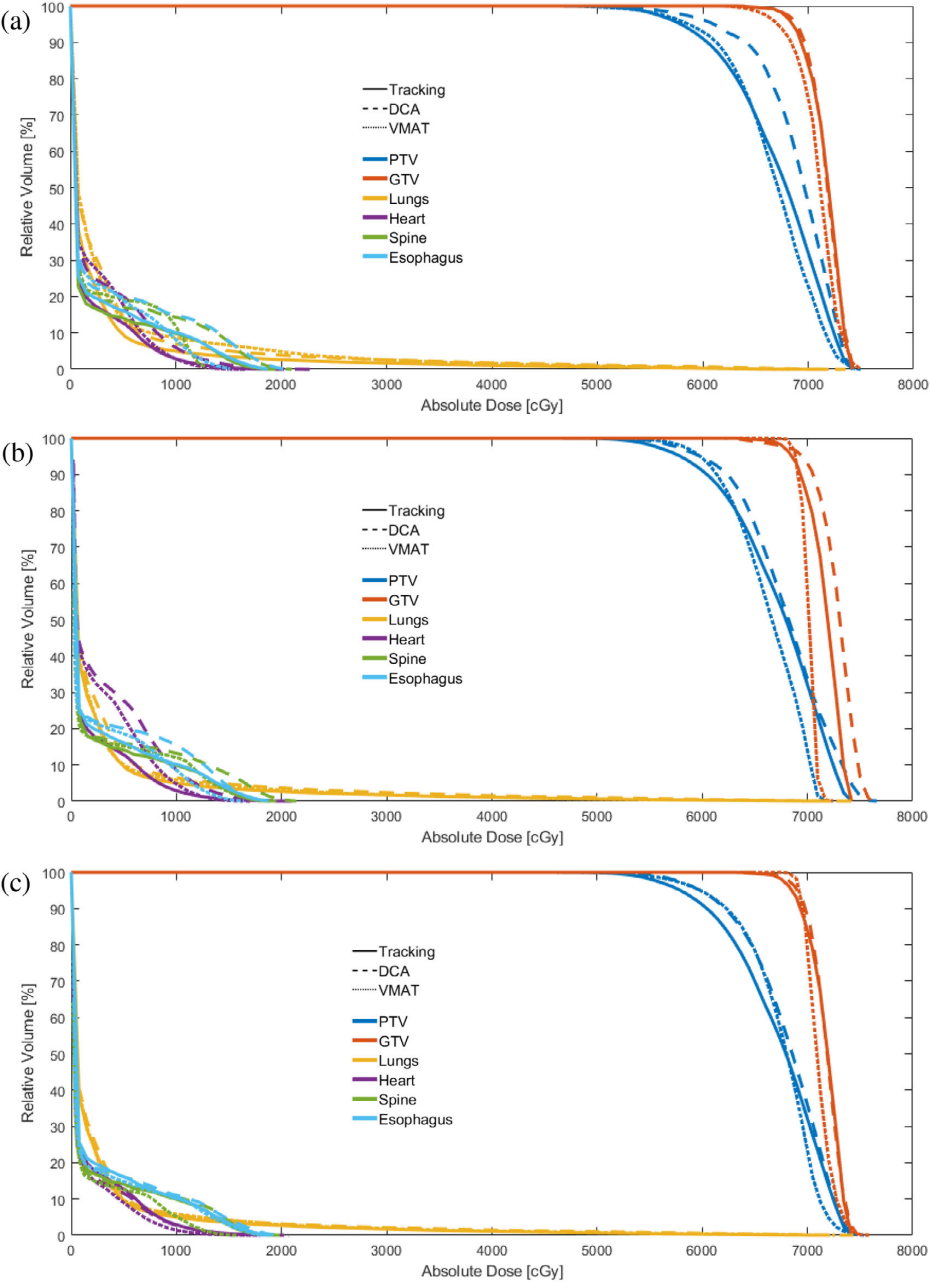
DVHs for a single sample patient comparing the tracking technique (solid line) to a DCA (dashed line) and VMAT (dotted line) technique utilizing an ITV‐based approach (a), a DIBH approach (b), and a gating approach (c). DCA, dynamic conformal arc; DIBH, deep inspiration breath hold; DVH, dose volume histograms; ITV, internal target volume; VMAT, volumetric modulated arc therapy.

DVHs for a sample patient are shown in Figure 3, comparing the performance of tracking to DCA and VMAT plans using each respiratory management technique. The ITV technique shown incorporates patient respiration with a 4D dose recalculation.

**FIGURE 4:**
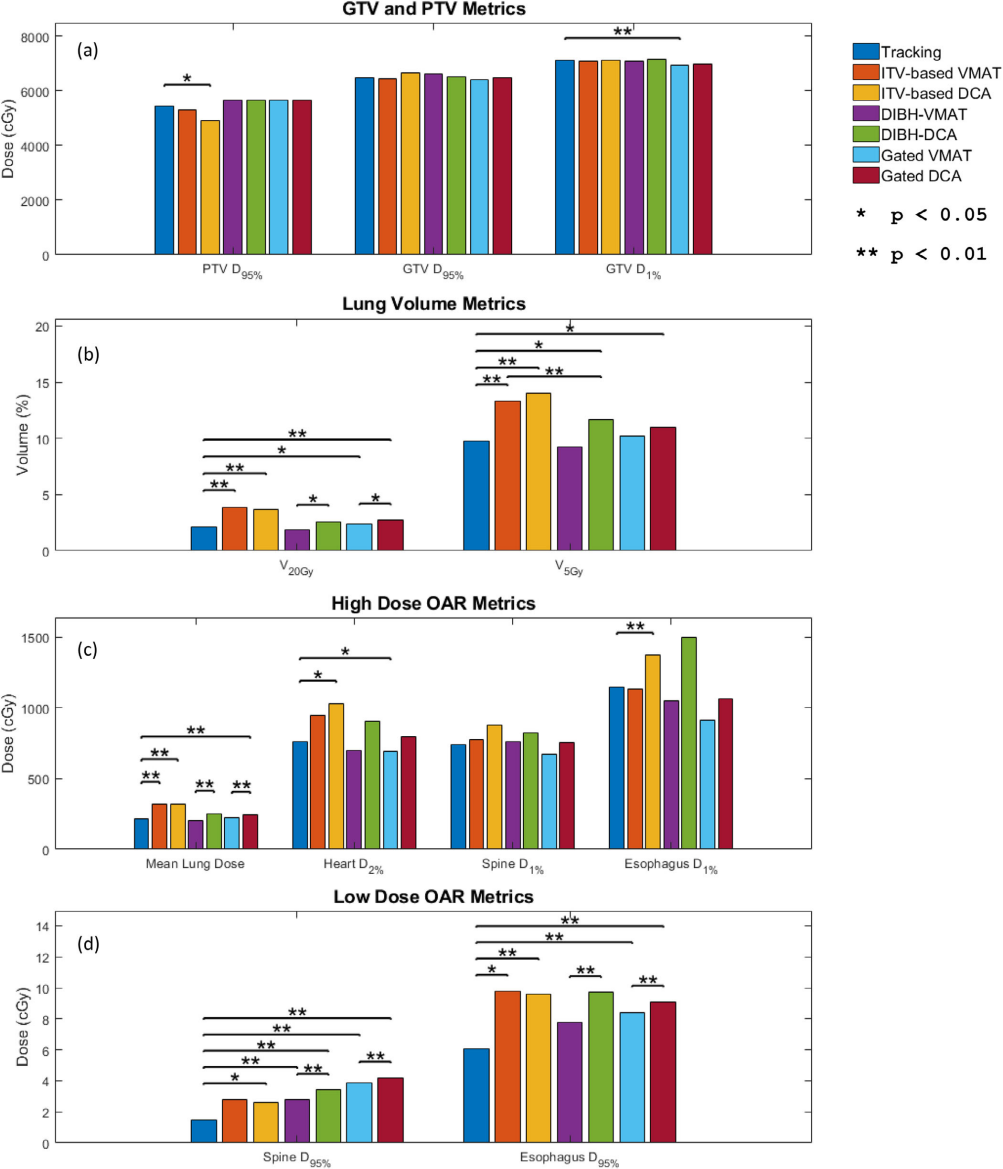
Comparison of different motion management techniques averaged over the patient population. Target coverage (a), lung volumes (b), and high and low OAR doses (c and d) were the reported DVH metrics. Pairwise comparisons were only reported if the overall (ANOVA) *p*‐value was significant and if the *p*‐value passes the Holm‐Bonferroni test. ANOVA, analysis of variance; DVH, dose volume histograms; OAR, organs at risk.

GTV coverage was within 5% of each other in Figure 3a–c. However, the target hotspot was greater with DIBH‐DCA compared to VMAT and tracking, although this increase was not statistically significant across the patient population (*p* > 0.05). In Figure 3a, the lung V_20Gy_ was reduced by over 35% using the DCA‐tracking technique relative to the VMAT or DCA technique using motion encompassment. Comparatively, in Figure 3b and 3c, lung V_20Gy_ was only decreased by 5%−7% when compared to VMAT and 11%−22% when compared to DCA. Averaged dose metrics and plan parameters for the tracking, DCA, and VMAT plans utilizing all three respiratory management techniques are summarized in Figure 4.

The tracking plans were able to obtain similar coverage as the VMAT plans using an ITV, while the DCA plans exhibited slightly reduced PTV coverage compared to the VMAT (*p* < 0.05). However, the GTV in each case received equal coverage in all three techniques with no significant difference (*p* > 0.05).

The tracking plans outperformed the ITV‐based DCA plan across all OAR metrics (*p* < 0.05), while it had superior or equivalent performance to the ITV‐based VMAT plans. While the tracking plan was able to reduce the minimum dose in the esophagus and spine compared to the VMAT plans (*p* < 0.05), the tracking plan maximum doses were not significantly different (*p* > 0.05). Similarly, the tracking did not show a significant reduction in maximum heart volumes compared to VMAT (*p* > 0.05).

Compared to the DIBH techniques, the tracking plans had reduced PTV coverage, but differences were not significant (*p* > 0.05). Additionally, GTV coverage and hotspots were comparable between the three techniques (*p* > 0.05). Differences in normal lung sparing were not statistically significant between the tracking and VMAT plans, while the DCA plans had greater V_20Gy_, V_5Gy_, and mean lung dose values (*p* < 0.05). Institutional clinical constraints were still met in all cases.

Spine, esophagus, and heart sparing at 1%−2% volumes were typically equivalent between the VMAT and tracking plans provided the GTV was not located near any OARs, while the DCA plan quality suffered. At 95% spine and esophagus volumes, VMAT and tracking outperformed DCA (*p* < 0.05).

When comparing the tracked plans to a respiratory gated technique, the tracking plan showed reduced PTV coverage, but differences were not significant (*p* > 0.05). GTV coverage was not statistically different between all three techniques, although the tracking plan did show a higher hotspot compared to both VMAT and DCA when using a gated technique (*p* < 0.05). DCA‐based tracking had a lower V_20Gy_ compared to gated VMAT and DCA (*p* < 0.05) and a lower V_5Gy_ and mean dose compared to gated DCA (*p* < 0.05). Differences in V_5Gy_ and mean dose between tracking and gated VMAT were not significant (*p* > 0.05). However, the VMAT technique did show reduced heart D_2%_ in the heart compared to tracking (*p* < 0.05).

Similar results were observed at larger and regional volumes, with D_1%_ values showing no significant difference after the post‐hoc correction, while the tracking plan has superior performance in sparing at 95% spine and esophagus volumes.

### 3.2 Treatment time comparisons

**FIGURE 5:**
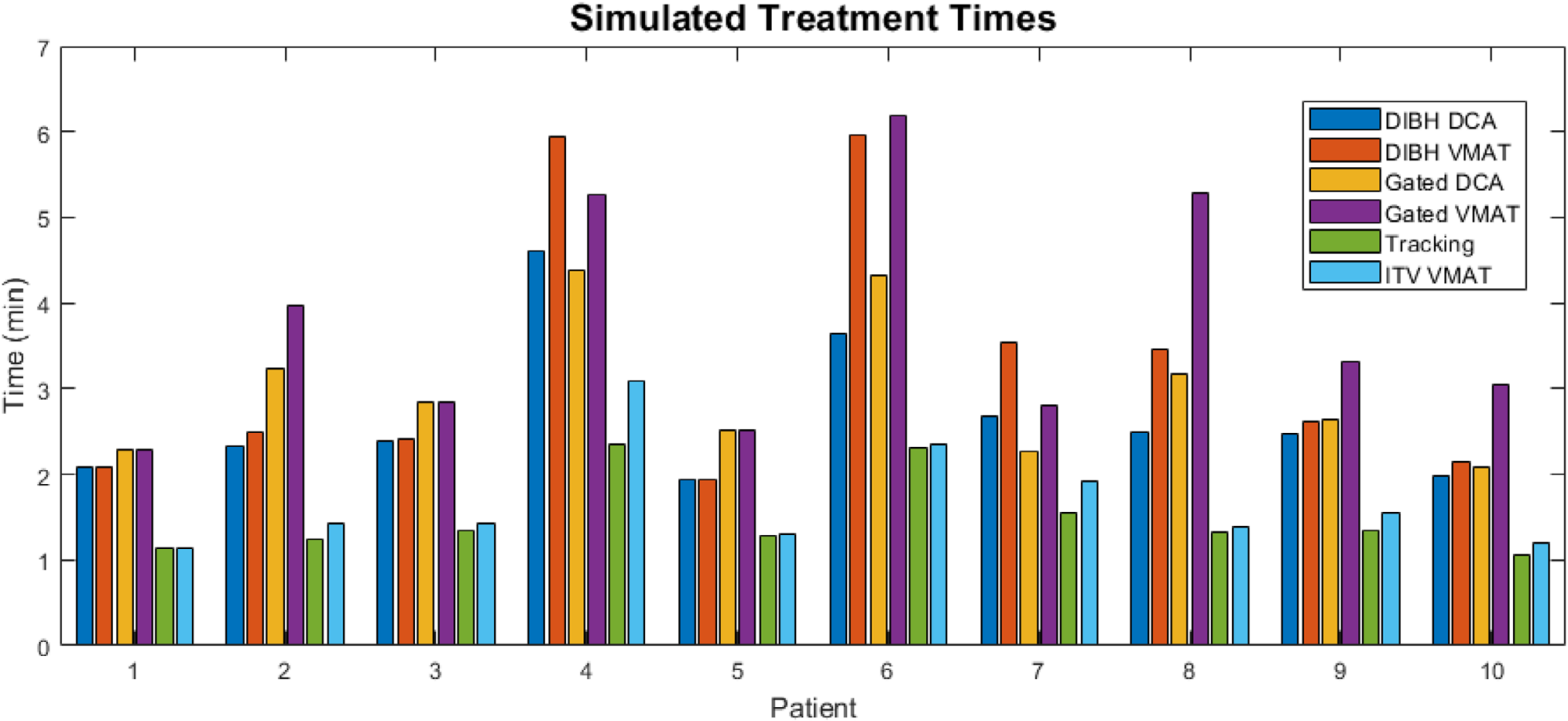
Simulated treatment times calculated from the beam‐on time for each treatment delivery and motion management technique pair for each patient.

Treatment times calculated from the beam‐on time of each plan are summarized in Figure 5. Tracking and ITV‐based DCA technique had identical treatment times, so no comparison was performed. A t‐test showed that the VMAT treatments were longer due to the modulation of the gantry speed (*p* < 0.05). Using a DIBH or gated technique showed treatment times significantly longer than the tracking technique (*p* < 0.01), with VMAT being longer than the corresponding DCA plan in both cases (*p* < 0.05).

### 3.3 4D dose calculation

**FIGURE 6:**
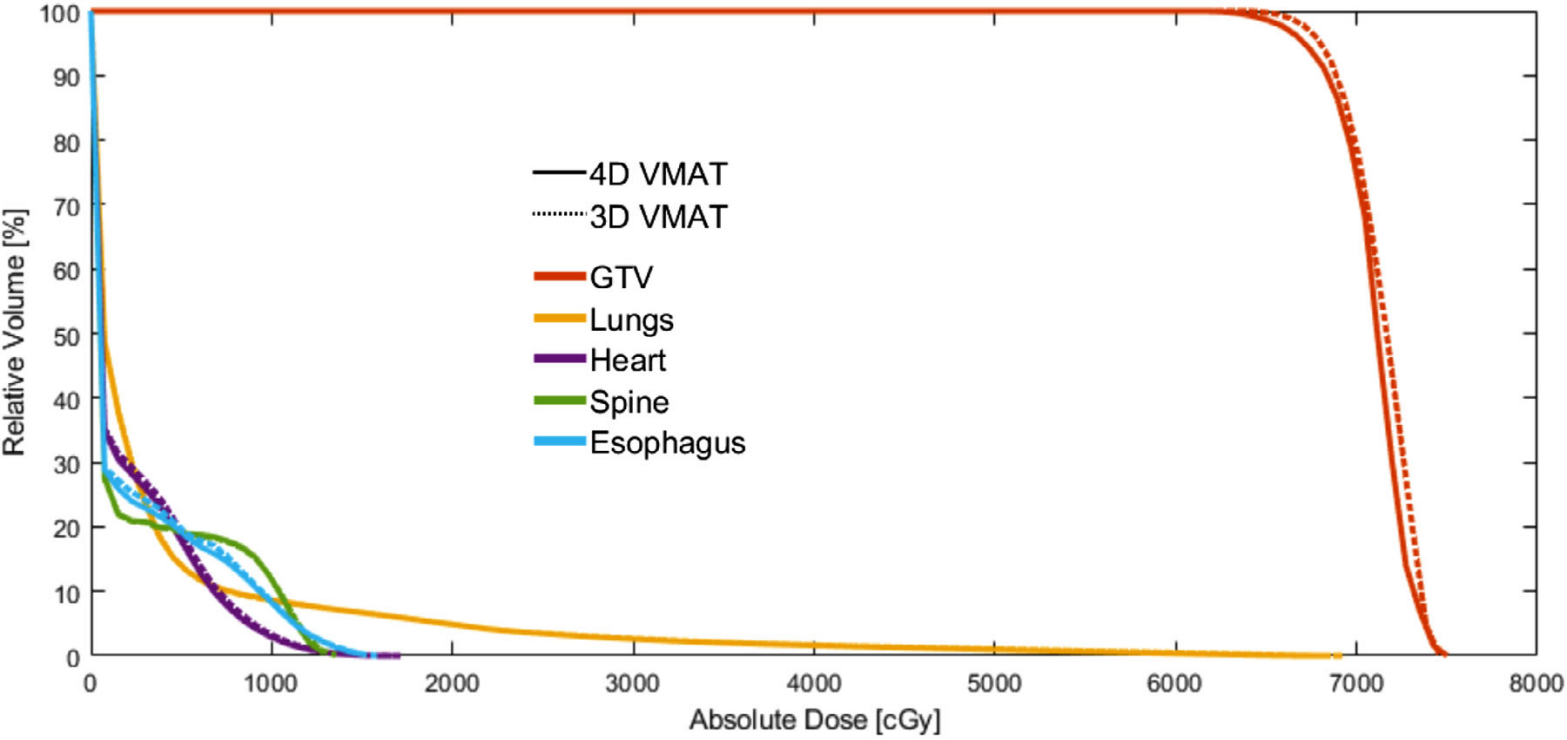
The same ITV‐based plan calculated once on the average free‐breathing CT and once over the entire 4D‐CT dataset using the 4D dose recalculation algorithm described in section B and C of the methods to account for respiratory‐related errors within an ITV technique. The 4D VMAT was just a recalculation over the 4D‐CT and was not reoptimized to account for respiratory motion. ITV, internal target volume; VMAT, volumetric modulated arc therapy.

DVHs for a sample patient's VMAT plan showing the change in GTV and OAR dose are show in Figure 6. The GTV DVH was slightly decreased for the sample patient in Figure 6, although the computed GTV metrics showed a change under 5%. Similarly, the OAR dose‐volume metrics all had changes under 5%. The effects of the 4D dose recalculation over the patient population are summarized in Table 3.

**TABLE 3:** Differences in GTV dose with the standard 3D calculation vs the recalculation of the full 4D‐CT. The 4D calculation algorithm for both the VMAT and DCA techniques directly accounts for the effects of patient respiration on GTV coverage during the ITV‐based treatments.

	3D	4D	Average percent difference	*p*‐value
DCA GTV D_95%_ (cGy)	6586.4	6632.6	0.6%	0.063
VMAT GTV D_95%_ (cGy)	6467.0	6429.9	−0.5%	0.31
DCA GTV D_1%_ (cGy)	7150.4	7112.9	−0.5%	0.012*
VMAT GTV D_1%_ (cGy)	7124.9	7079.4	−0.6%	0.085

Abbreviations: DCA, dynamic conformal arc; GTV, gross tumor volume; ITV, internal target volume; VMAT, volumetric modulated arc therapy.

The 4D recalculation did not show any statistically significant changes in the GTV coverage caused by the interplay effect for the VMAT plans. The DCA plans showed a statistically significant increase to the GTV D_1%_ value (*p* < 0.05), although the average change was under 1% and all patient‐specific changes were under 5%.

## 4 DISCUSSION

This study compared the performance of a DCA‐based tracking technique to other commonly implemented respiratory management techniques using VMAT and DCA. Similar dosimetric studies comparing the performance of tracking techniques to other motion management methods have been conducted, although none have focused on implementing DCA with tracking. Instead, commonly implemented tracking techniques tend to use VMAT,^32^, ^33^, ^34^, ^35^ intensity modulated radiation therapy (IMRT),^36^, ^37^, ^38^ or fixed field 3D conformal radiation therapy (3D‐CRT).^38^, ^39^ The AAPM task group 264^40^ gives a concise summary of the current progress on clinically implementing DMLC tracking and comments on how most work has focused on VMAT as the primary modality for implementing DMLC tracking.

Beyond dosimetric studies, the feasibility of using DCA with a tracking technique has been previously considered in the literature. Jones et al.^41^ has investigated a scatter imaging‐based tracking technique that can be applicable with DCA, but not with VMAT due to the increased modulation reducing accuracy. Xu et al.^42^ instead investigated the optimal collimator and couch angle for DCA tracking to reduce tracking complexity and improve plan robustness. As such, there is an absence of treatment planning studies using tracked DCA plans. To the authors’ best knowledge, this study represents the first time that a DCA tracking technique has been analyzed in a treatment planning setting.

While ITV‐based methods for motion compensation are less time intensive than gated and breath‐hold techniques by allowing the patient to breathe freely during treatment, their performance relative to other motion management techniques can vary. Most studies indicate that using gated and DIBH treatments where possible is preferable,^43^, ^44^ although when PTV volumes are sufficiently small, ITV methods can have comparable performance to gating.^45^ Tracking with DCA showed similar results, where a tracking technique outperformed the ITV technique across all patients in normal lung sparing. Despite the fewer degrees of freedom for optimization in the tracked DCA plans, the reduction in treatment margins when moving from an ITV to a GTV were sufficient to significantly reduce the normal lung dose compared to VMAT. The effect of reducing treatment margins is further emphasized when we consider the change in low dose spillage to OARs seen in the D_95%_ value. The DCA‐tracking plans showed lower D_95%_ values in the spine compared to all other treatment combinations, including VMAT. VMAT optimization guidelines for the spine typically aim to reduce the maximum dose or the dose to a small volume of the spine (e.g., 5 cc). As a result, most VMAT optimizations do not prioritize reducing the low dose delivered to 95% of the spine volume and the tracked plans were able to demonstrate a significantly lower D_95%_ value due to the reduced treatment margins. Whether this reduction in D_95%_ is clinically significant warrants future study and is beyond the scope of the current treatment planning study.

Comparisons between a tracking technique with gating and DIBH, which are similar on a single‐phase basis, yielded more comparable dose‐volume metrics in the normal lung. The non‐tracked DCA plans tended to perform worse than their VMAT counterparts due to the absence of modulation to control the presence of a hotspot within the GTV. While an inverse‐optimized VMAT plan might be able to modulate several parameters to control for the GTV dose, the forward‐planned DCA plans were only capable of adjusting the treatment margins to account for a hotspot on a patient‐specific basis, increasing the normal lung dose. Additionally, on a DIBH image, there may be an increased low‐density environment around the GTV that could widen the beam penumbra,^46^ even when using 6MV x‐rays, that would need to be further accounted for by increasing the margin in the DCA plans.

With regards to treatment time, each treatment plan analyzed in this study takes around 1−2 min to deliver while assuming continuous irradiation. As a result, several breath‐holds would be required to deliver the full beam, increasing treatment time with a DIBH technique. Additionally, gated plans can potentially triple the required treatment time depending on the gating window used. As a result, the simulated DIBH and gated delivery times in this study are all several minutes longer than the tracked plans. It should be noted that while the breath hold window used for treatment time calculation in the DIBH plans were derived from their DIBH CT scan, it is possible that some patients could hold their breaths longer than the required duration for the DIBH CT scan. As such, our method overestimates the DIBH treatment time. However, the time‐saving benefits of tracking over DIBH are still presented should the patient require even a single recovery period during treatment, which is highly likely for treatment plans taking over 2 min to deliver with continuous irradiation.

With shorter beam‐on times, patient time on the treatment couch can be minimized, reducing the likelihood of patient movement during treatment. Given that the tracked plans showed similar dosimetric performance to the gated and DIBH plans, the additional time‐saving benefits makes tracking an ideal motion management technique. While the bulk of time‐savings are derived from reducing the dead‐time present in gated and DIBH deliveries, the VMAT plans were still found to require more time to deliver due to the varying gantry speed during treatment, indicating the simpler delivery method was capable of maximizing the time‐saving benefits.

However, the absence of modulation limited the amount of sparing present in other OARs in some cases. While the reduction in margins has benefits to the global structures that the tumor was embedded in, such as the lung, the absence of modulation prevents the tracked DCA plan from optimally sparing OARs if they enter the beam's aperture compared to VMAT, such as with the esophagus and spine. This is further seen when comparing the change in D_95%_ and D_1%_ in the spine and esophagus. The smaller treatment fields resulted in a significant reduction in the low dose spilling to the OARs, but the absence of modulation could not limit the higher dose from being deposited. Although, for most cases, this increase in dose is still well within clinical tolerances.

**FIGURE 7:**
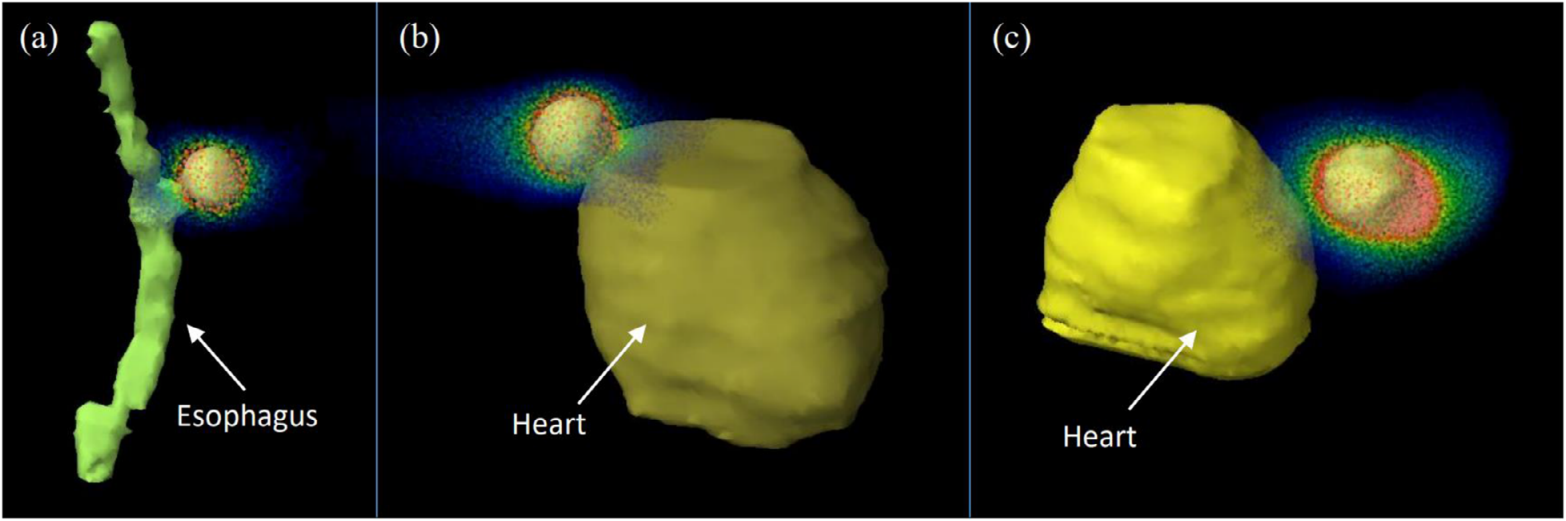
The dose distribution for three patients with the PTV near the esophagus (a) or heart (b and c). The dose distribution is displayed from 15 Gy up to the prescription dose covering the PTV. Close proximity with the PTV can result in a significantly higher dose to the OAR that a DCA technique might be unable to effectively reduce. DCA, dynamic conformal arc; OAR, organs at risk.

In more complex cases, this issue can be further amplified when the PTV is located near a sensitive OAR, with one case even seeing a 107% hotspot in the esophagus in a DIBH delivery. In patient A, shown in Figure 7, the GTV was located nearby the esophagus, with the deep inhalation moving the GTV even closer to the esophagus. Here, the forward‐planned DIBH‐DCA plan resulted in a higher OAR dose, skewing the esophagus D_1%_ average shown in Figure 4c. In such cases, VMAT can be a more optimal technique since the increased modulation available allows for the delivery of more conformal dose distributions that can effectively carve the dose out of the surrounding OAR. For tracked DCA, even with the reduced treatment margin, close proximity of the OAR results in a significantly higher dose that can exceed clinical constraints depending on the prescription and OAR location. Three such cases highlight where the PTV was located next to sensitive OARs in Figure 7 and are summarized in Table 4. When comparing the high dose spillage into these structures across all treatment techniques and motion management strategies, gated and DIBH VMAT showed the greatest OAR sparing. As such, a DCA‐based tracking technique is more appropriate when treating tumor locations where OARs are less likely to enter the open radiation field.

**TABLE 4:** Exceeded dose constraints for three sample patients. When the OAR is near the PTV, dose spillage can exceed dose constraints at regional volumes. The increased modulation present in VMAT is able to more effectively reduce the max dose in such cases.

	Esophagus D_1%_ (cGy)	Heart D_1%_ (cGy)		Heart D_2%_ (cGy)	
Treatment technique	Patient A	Patient B	Patient C	Patient B	Patient C
Tracking	3756.4	2827.1	2129.1	1921.2	1850.1
ITV VMAT	3530.3	3338.1	2002.9	2408.4	1796.1
ITV DCA	4491.9	3815.3	2540.3	2526.4	2228.4
Gated VMAT	2492.1	2422.7	2006.9	1722.6	1722.8
Gated DCA	2698.2	2856.9	2260.4	1934.6	1963.4
DIBH VMAT	3525.0	2110.4	1728.5	1579.8	1526.0
DIBH DCA	6414.8	3446.5	1904.8	2484.9	1699.0

Abbreviations: DCA, dynamic conformal arc; DIBH, deep inspiration breath hold; ITV, internal target volume; OAR, organs at risk; VMAT, volumetric modulated arc therapy.

In non‐tracking settings, some studies have shown that DCA can achieve acceptable plans for lung SBRT.^21^, ^22^, ^24^ Additionally, some have proposed that even if conventional forward‐planned techniques underperform relative to VMAT and IMRT, they exhibit enough benefits that they should remain as viable alternatives in centers without inverse planning capabilities.^24^, ^47^ Conventional 3D treatment techniques, including DCA, are easier to perform quality assurance, faster to deliver, and less susceptible to dose errors such as the interplay effect compared to VMAT. While this cohort of patients did not exhibit a clinically significant interplay effect, as shown in Table 3, this is not universally true with all VMAT plans.^12^, ^16^, ^17^ Comparatively, a DCA delivery where the tumor is never covered by moving MLCs during treatment would have a smaller contribution to the interplay effect due to the reduced MLC modulation and uniform fluence to the GTV, which is a significant consideration when introducing an additional degree of freedom through tracking. However, an interplay effect resulting from varying gantry speed, dose rate, or tracking latency could still be present. When implementing a tracking approach, DCA would be able to maintain those advantages over VMAT and IMRT while having a comparable dosimetric performance with DIBH and gated VMAT plans. Additionally, while the dosimetric performance of the tracked DCA plan is comparable to VMAT with other techniques, the tracking plan is significantly faster to deliver due to the near 100% duty cycle, unlike gating and DIBH which would also further require patient compliance for viability. These times are even further extended when considering fixed‐field techniques, such as with IMRT or CyberKnife. Zyp et al.^48^ reported that lung SBRT treatments using a CyberKnife had a median treatment time of 100 min, while a tracked DCA plan could be delivered on the order of a few minutes. The novelty of this technique is further emphasized when we consider that hybrid MR‐Linear Accelerators, which are a promising modality for real‐time tumor tracking, are currently incapable of delivering arc‐based therapies, necessarily lengthening treatment times.^49^, ^50^

The current study is still limited by its small sample size. Some results showed significance after performing an ANOVA test but failed the post‐hoc correction, possibly indicating that a greater sample size is required. Furthermore, our tracking dose calculation algorithm relies on the use of dose deformation algorithms that represents a degree of uncertainty not considered in this study. Nelli et al.^30^ conducted a similar single‐phase 4D‐CT dose accumulation study to analyze the impact of respiratory motion on a VMAT SBRT treatment and found that the dose deformation algorithm performed exceptionally well, with a 99.2% and 99.1% minimum pass rate when considering a motion amplitude of 1  or 2 cm respectively. However, while quality assurance of patient‐specific plans accounting for patient‐specific respiratory curves and anatomy should be considered prior to clinical testing, they remain beyond the scope of this current study.

Future work would focus on the accuracy of the dose deformation evaluated on a patient‐specific basis. Additionally, this treatment planning study assumes idealized deliveries without considering the effects of MLC latency or tracking errors. However, VMAT tracking has already been shown to be viable so long as MLC latency is less than 150 ms^34^ and the reduced modulation in DCA should also reduce the impact of machine latency during tracking. Future work analyzing the impact of tracking latency should be considered once the chosen tracking algorithm is established and inherent latency quantified.

## 5 CONCLUSION

DMLC tracking with DCA has dosimetric and time‐saving benefits compared to VMAT plans delivered with other respiratory management techniques, while allowing patients to breathe freely. The reduction in treatment margins can significantly reduce the normal lung dose compared to ITV techniques and has comparable performance to gating and DIBH while significantly reducing treatment times. In cases where lung tumors located away from critical OARs exhibit large respiratory motion, DMLC tracking with DCA could represent an ideal treatment alternative to existing motion management techniques.

## AUTHOR CONTRIBUTIONS

Timothy Yau contributed to the study design, data collection, analysis, and interpretation of data, drafted the manuscript, and approved the final version. Jeff Kempe contributed to the study design, analysis of data, and approved the final version. Stewart Gaede contributed to the study design, study supervision, data analysis and interpretation, contributed to drafting of the manuscript, approved the final version, and funded the project.

## CONFLICT OF INTEREST STATEMENT

The authors declare no conflicts of interest.

## Data Availability

The data that support the findings of this study are available on request from the corresponding author. The data is not publicly available due to privacy or ethical restrictions.
